# **Tracking magma pathways and surface faulting in the Southwest Rift Zone and the Koa**ʻ**e fault system (Kīlauea volcano, Hawai ‘i) using photogrammetry and structural observations**

**DOI:** 10.1007/s00445-024-01735-7

**Published:** 2024-04-11

**Authors:** Stefano Mannini, Joël Ruch, Richard W. Hazlett, Drew T. Downs, Carolyn E. Parcheta, Steven P. Lundblad, James L. Anderson, Ryan Perroy, Nicolas Oestreicher

**Affiliations:** 1 Department of Earth Sciences, University of Geneva, Rue Des Maraîchers 13, 1205 Geneva, Switzerland; 2 Department of Geology, University of Hawai‘I at Hilo, Hilo, HI 96720 USA; 3 U.S. Geological Survey, Hawaiian Volcano Observatory, Hilo, HI 96720 USA; 4 Alaska Earthquake Center, University of Alaska Fairbanks, Fairbanks, AK 99775 USA; 5 Department of Geography and Environmental Science, University of Hawai‘I at Hilo, Hilo, HI 96720 USA

**Keywords:** Faulting, Magma propagation, Ground deformation, Monocline, Kīlauea volcano

## Abstract

Volcanic islands are often subject to flank instability, resulting from a combination of magmatic intrusions along rift zones and gravitational spreading causing extensional faulting at the surface. Here, we study the Koaʻe fault system (KFS), located south of the summit caldera of Kīlauea volcano in Hawaiʻi, one of the most active volcanoes on Earth, prone to active faulting, episodic dike intrusions, and flank instability. Two rift zones and the KFS are major structures controlling volcanic flank instability and magma propagation. Although several magmatic intrusions occurred over the KFS, the link between these faults, two nearby rift zones and the flank instability, is still poorly studied. To better characterize the KFS and its structural linkage with the surrounding fault and rift zones, we performed a detailed structural analysis of the extensional fault system, coupled with a helicopter photogrammetric survey, covering part of the south flank of Kīlauea. We generated a high-resolution DEM (~ 8 cm) and orthomosaic (~ 4 cm) to map the fracture field in detail. We also collected ~ 1000 ground structural measurements of extensional fractures during our three field missions (2019, 2022, and 2023). We observed many small, interconnected grabens, monoclines, rollover structures, and en-echelon fractures that were in part previously undocumented. We estimate the cumulative displacement rate across the KFS during the last 600 ~ 700 years and found a decrease toward the west of the horizontal component from 2 to 6 cm per year, consistent with GNSS data. Integrating morphology observations, fault mapping, and kinematic measurements, we propose a new kinematic model of the upper part of the Kīlauea’s south flank, suggesting a clockwise rotation and a translation of a triangular wedge. This wedge is bordered by the extensional structures (ERZ, SWRZ, and the KFS), largely influenced by gravitational spreading. These findings illustrate a structural linkage between the two rift zones and the KFS, the latter being episodically affected by dike intrusions.

**Supplementary Information:**

The online version contains supplementary material available at 10.1007/s00445-024-01735-7.

## Introduction

Normal fault systems, eruptive fissures, and grabens in volcanically active zones are often the surface expression of subsurface dikes and magma pathways. There are several basaltic volcanic areas on Earth where such volcano-related structures are notably well-developed, such as the Hawaiian volcanoes (USA, Peacock and Parfitt 2002), Etna (Italy, Neri and Acocella 2006), the Afar region (Ethiopia, Acocella et al. 2003), and along rift zones in Iceland (Gudmundsson and Bäckström, 1991). These normal faults are accompanied by extensional fractures, monoclines, buckles, and vertical scarps (Holland et al. 2006), which can be frequently reactivated during earthquakes or magmatic intrusions (Rivalta et al. 2015; Sigmundsson et al. 2015; Ruch et al. 2016). However, the temporal and spatial relationship between these processes in shaping the south flank is still poorly understood.

In this study, we address the importance of these volcano-tectonic features to deepen our understanding on magma propagation and tectonic stability in a rapidly growing volcano. We focused our analysis on Kīlauea volcano (Hawai ‘i) which is characterized by recurrent volcano-tectonic events and has well-documented normal faulting over its south flank (Swanson et al. 1976; Denlinger and Okubo 1995; Owen et al. 1995; Montgomery‐Brown et al. 2015; Swanson et al. 2018). The summit caldera is connected to two rift zones (East and Southwest Rift Zones; ERZ and SWRZ, respectively) that are presumed to be structurally linked by the Koaʻe fault system (KFS) (Duffield 1975). The KFS is characterized by a series of normal faults with a dominant extensional component. The development of the KFS is still taking place as the whole of Kīlauea’s southern flank moves seaward, as shown by widespread submarine faulting along the Hilina and Punaluʻu slumps (Morgan et al. 2003). In contrast, the region north of the summit caldera and rift zones remains stable because it is buttressed by Mauna Loa’s edifice (Lipman et al. 2006).

**Fig. 1:**
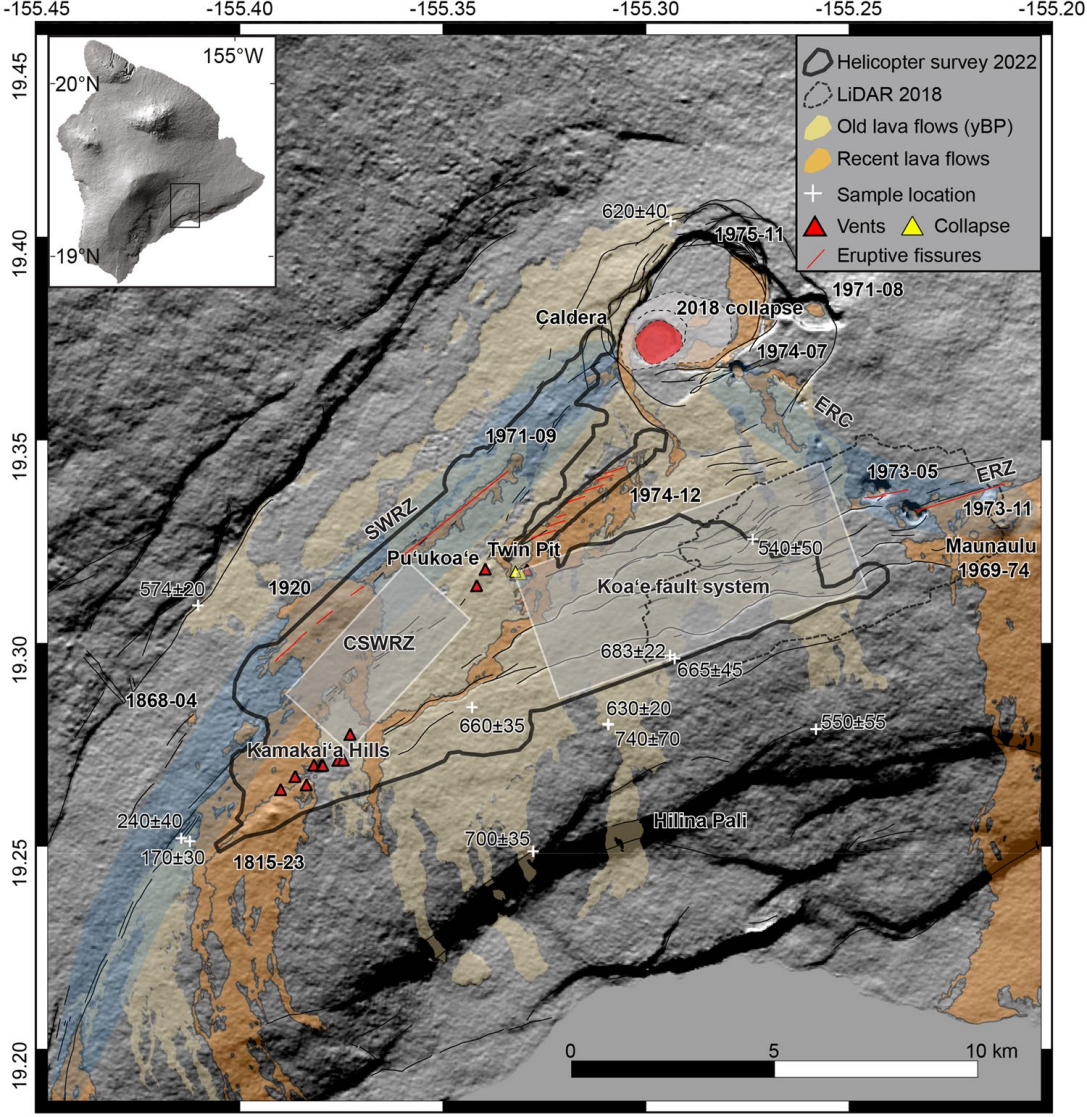
Digital elevation model (30 m) overlain by the principal key structures and lava flow ages of Kīlauea’s south flank. Recent lava flows (last two centuries) are depicted in orange, and lava flows 200–800 years old in light orange. White crosses are the locations of radiocarbon ages, red triangles are scoria cones, and red dashed lines are eruptive fissures. ERC, East Rift connector (Swanson et al. 2018); ERZ, East Rift Zone; SWRZ, Southwest Rift Zone; and CSWRZ, central Southwest Rift Zone are highlighted in transparent blue. The black polygon shows the area covered by our helicopter survey in 2022 and the thin gray dashed line by the 2018 LiDAR mission. White rectangles are the regions of study

During the last century, dozens of volcano-tectonic events occurred along the south flank of Kīlauea, some of which accompanied eruptions at the summit and in the rift zones (Fig. 1) (Swanson et al. 1976; Montgomery‐Brown et al. 2010; Poland et al. 2014; Montgomery-Brown and Miklius 2021). Of the two rift zones, the ERZ is the most active and the KFS defines the westward structural continuation of the ERZ. The KFS dilates parallel to the ERZ along this alignment, forming a network of faults that reactivate during earthquakes and flank movement. In addition to regional tectonics, which also explains extension across the ERZ, intrusions from the rift zone have penetrated the eastern end of the KFS, such as during the episode of May 1973 (Swanson et al. 2018) following pre-existing structures.

Structures in the KFS remain well exposed and preserved, compared to structures located along the rift zones that are covered by recent lava flows. Notably, the KFS and the entire south flank have been well-monitored since 1966 using EDM (electronic distance measurer) and leveling measurements, showing variations in inflation and deflation over the area (Swanson et al. 1976). Here, we aim to map in detail the features well-exposed in the central SWRZ, KFS, and ERZ in the vicinity of the Maunaulu (Fig. 1) to better understand the interaction between magmatism and tectonism.

Based upon our field observations, we highlight the evidence of magma pathways, defined as a magma corridor, and estimate the amount of cumulative deformation over the study area (Fig. 1). We also propose a kinematic model for the upper flank of Kīlauea, south of the summit caldera—the thickest part of the shield. Our model specifically encompasses a triangular wedge bounded by the summit caldera, the SWRZ and ERZ, and the southern part of the KFS. Specific geographical areas of interest are described in the following subsections.

### Koaʻe fault system

**Fig. 2:**
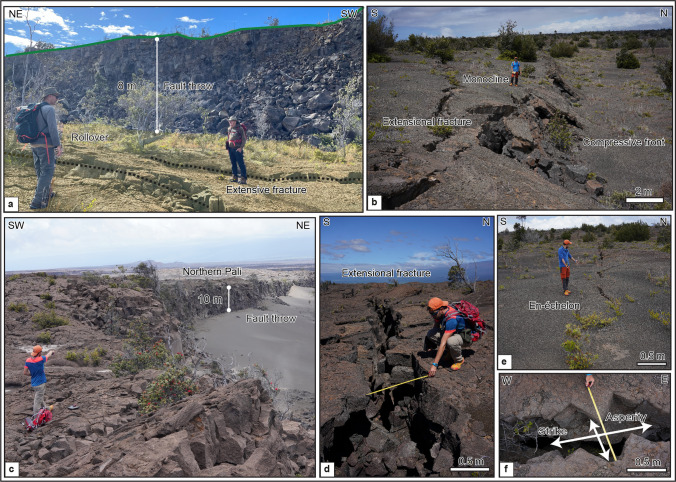
**a** Kulanaokuaiki Pali showing vertical displacement with a fault throw of ~ 8 m, and centimeter extensional fractures and prominent rollover features at its base. **b** Ohale Pali highlights a monocline with extensional fractures located at its top, and a compressional front at the bottom. **c** Northern pali where the Koaʻe fault system has a ~ 10-m vertical offset. **d** Example of extensional fracture measurement. **e** Example of centimeter en-echelon ground cracks. **f** Example of kinematic measurements showing the asperity fit and strike. See Figs. 6 and 7 for locations

The KFS is located south of the summit caldera and between the ERZ and SWRZ. It defines the northern edge of the Kīlauea’s south flank (Fig. 1). This fault system covers an area ~ 12 km long by 3 km wide, with most fault scarp facing to the north (Duffield 1975). The principal structures visible over this fault system are typically normal faults and associated extensional fractures, monoclines, nested grabens, rollovers, and buckles (e.g., Holland et al. 2006; Martel and Langley 2006; Kaven and Martel 2007; Bubeck et al. 2018). In the KFS, normal faults are generally oriented ENE-WSW and E-W, forming nested graben structures that we interpret as forming during dike emplacement into Kīlauea’s rift zones. En-echelon structures and extensional fractures dominate this area and are of key importance for quantifying long-term deformation (Fig. 2).

These structures are defined as follows: extensional fractures are open cracks, and buckles are geological features formed by folding (Fossen 2016). A rollover (Fig. 2a) is a fold structure related to a listric normal fault with bedding in the downthrown block bent downward toward the fault plane (Xiao and Suppe 1992). Monoclines, common throughout the KFS, are usually associated with step-like folds; however, in the KFS, many monoclines form unruptured ramps along faults between a footwall and a hanging wall (Fig. 2a–c). In the literature, these are also called monoclinal flexures (Duffield 1975), tilted hanging walls (Trippanera et al. 2015), rotating blocks (van Gent et al. 2010), tilted limb of original monoclinal flexures (Holland et al. 2006), and tilted blocks (Angelier et al. 1997; Kettermann et al. 2019). Their geometries and relative formations are well explained in previous studies concerning the successive stages of normal fault growth (Holland et al. 2006; Martel and Langley 2006; Kaven and Martel 2007; Bubeck et al. 2018).

All of the lava flows mapped within the KFS are of tholeiitic basalt composition, with most emplaced during a period of extensive effusive lava flows onto the south flank from a shield that had infilled the older caldera (Wolfe and Morris 1996; Neal and Lockwood 2003; Sherrod et al. 2021; Sinton and Sherrod 2021). These predominantly pāhoehoe lava flows with minor ‘a ‘ā lava have been bracketed in age to an approximately 200-year time interval 600–700 years before present (Rubin et al. 1987; Wolfe and Morris 1996; Swanson et al. 2014; Reimer et al. 2020). Younger flows that erupted from the rift zones bound the margins of the KFS, such as the December 1974 eruption from the SWRZ that was emplaced in southerly and westerly directions following fault scarps of the KFS (Fig. 1). The summit flows that cover most of the KFS are notably older and lumped into four groups. We use the lava flow contacts of Sherrod et al. (2021) to discriminate between different Observatory vent lava flows, all of which they labeled as unit Qp4o. For ease of distinguishing and discussing these lava flows, we opt to use a modified nomenclature from Neal and Lockwood (2003) of lava flows of Luamanu, lava flows of Āhua, and younger lava flows of the Observatory vent (Figure S1).

Calibration of previous and new radiocarbon ages using Reimer et al. (2020) has allowed us to revise ages and create weighted mean ages of these lava flows, with only radiocarbon ages that are statistically the same at 95% confidence used for calculating weight mean ages. All these lava flow groups overlap in age, indicating that lava flow inundation of the KFS occurred throughout the thirteenth to fifteenth centuries (Table 1).

**Table 1:**
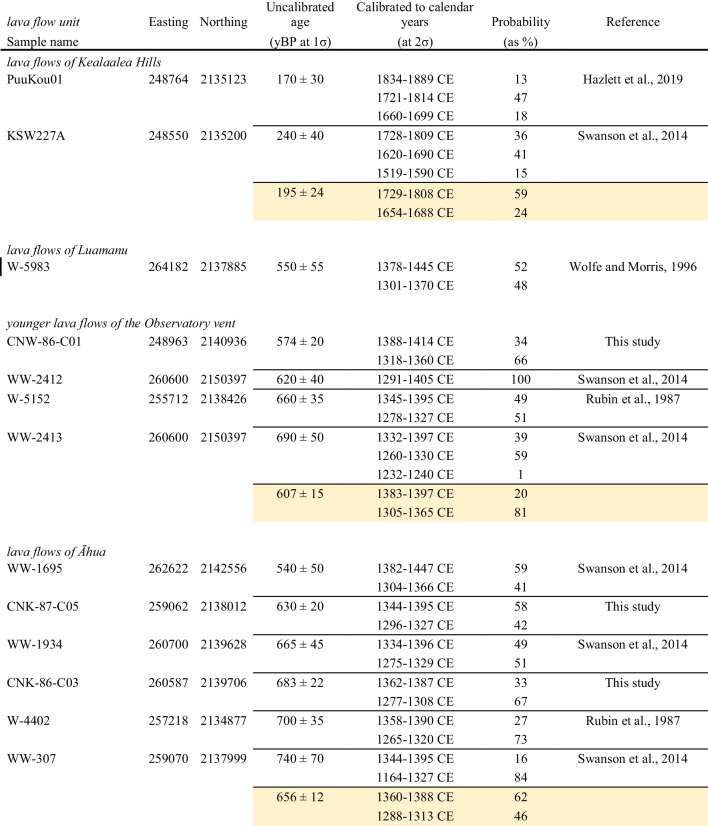
Radiocarbon ages from selected lava flows that cover the Koaʻe fault system and central Southwest Rift Zone of Kīlauea volcano

Note: All analyses were calibrated using calib rev 8.1.0 after Reimer et al. (2020). Uncalibrated ages are presented as years before present (yBP) with errors at 1σ uncertainties and calibrated ages are presented as calendar years as CE with errors at 2σ uncertainties. Light oranges are weighted mean ages using analyses that are statistically equivalent at 95% confidence. The lava flows of Kealaalea Hills age are further stratigraphically constrained to between 1790 and 1823 AD (Hazlett et al. 2019). Easting and northing coordinates are in NAD83 UTM zone 5.

### Central part of the Southwest Rift Zone

This area is part of the SWRZ and is bounded to the south by the 1919–1920 Maunaiki lava flows, to the west by the September 1971 lava flows, and to the east and north by the December 1974 lava flows (Fig. 1). This covers an area ~ 5 km long and 2 km wide and has major fault scarps enclosing a central graben bounded by the KFS to the south. These structures are oriented ENE-WSW and are more extensively covered by lava flows than those of the KFS, due to inundation by more recent and frequent eruptions. However, several faults and extensional fractures forming grabens are visible here and allow acquiring measurements over the central part of the SWRZ.

The SWRZ trends SW-SSW for ~ 30 km from the summit of Kīlauea to the coast, with a width ranging from 1 to 5 km. Since the catastrophic summit eruption of the 1790 CE Keanakākoʻi Tephra units I and J (Swanson and Houghton 2018), the SWRZ has erupted at least seven times over the past 200 years with the most recent eruption occurring in December 1974 (Neal and Lockwood 2003; Hazlett et al. 2019; Sherrod et al. 2021). Lava flows emplaced within the SWRZ are sourced from summit dike intrusions that erupt from vents, producing scoria cones and fissures spanning tens of kilometers. Lava flow compositions within the SWRZ are like those of the KFS, with tholeiitic basalts being ubiquitous (Wolfe and Morris 1996; Sherrod et al. 2021). Unlike lava flows in the KFS, though, a line of scoria cones termed the Kamakaiʻa Hills within the central part of the SWRZ have anomalously high-SiO_2_ and low-MgO compositions, equating to basaltic andesite (Fig. 1) (Hazlett et al. 2019; Downs et al. 2023).

## Methods

Surface structures located within the study area were mapped using high-resolution (5–10 cm) optical images acquired by a helicopter survey and complemented by field observations. Combining remote sensing with ground observations has allowed us to analyze the same features at different scales and, thus, to better understand their origin and formation. During the 2018 LiDAR flights, the U.S. Geological Survey also acquired high-resolution images (5 cm) over the eastern part of the KFS (Fig. 1). However, we are interested in the entire KFS, so we decided to extend the data set of the 2018 LiDAR mission acquiring more data over the area for this study.

**Fig. 3:**
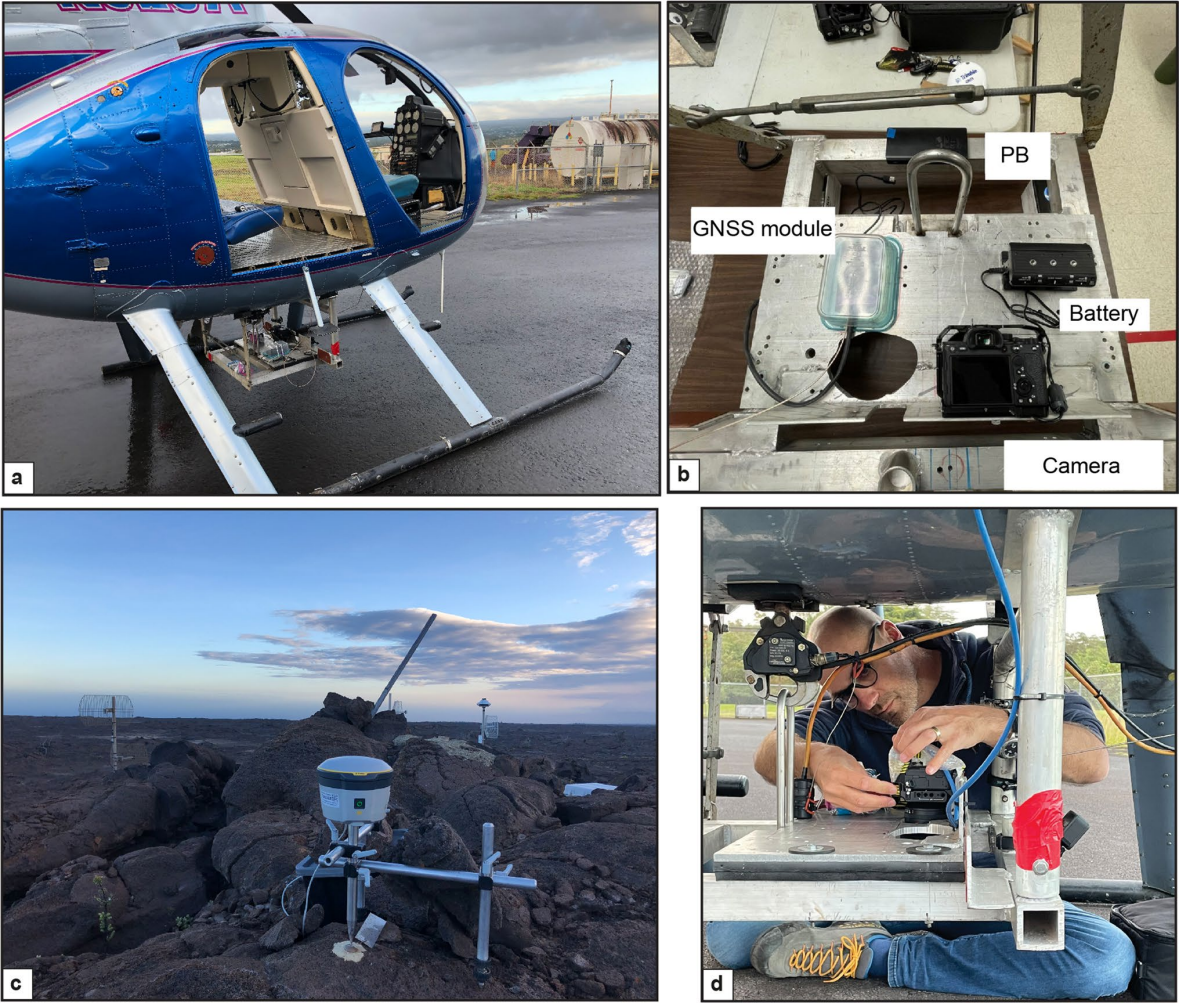
Photogrammetric survey setup with **a** Helicopter Hughes 500. **b** Payload developed by Perroy et al. (2021) with Sony Alpha7 RIV and Nikon D850 DSLR cameras, a GNSS module and antenna (Emlid Reach M2), powered by a power bank (PB). **c** Trimble® R2 GNSS receiver as base station for image orthorectification. **d** Setting the camera under the helicopter before the take-off

Photogrammetry surveys were performed using a Hughes 500 MD369C helicopter (Fig. 3a) carrying a custom imaging payload developed by the University of Hawaiʻi at Hilo Spatial Data Analysis & Visualization (SDAV) Research laboratory in partnership with the National Park Service, Volcano Helicopters, and R&R Machining and Welding in Hilo, Hawaiʻi (Perroy et al. 2021). The modular camera configuration included a Sony Alpha7 RIV camera (61 MPx) with a Sony GM 35 mm f/1.5 lens, a Nikon D850 DSLR camera (FX-format full-frame CMOS, 45.7 MP) a GNSS system for precise photo geotagging purposes (Fig. 3). The surveys were carried out during April 2022 at a flying altitude of 400 m above the ground surface, with each survey lasting for around 60–70 min. The acquired images were geotagged with a GNSS system on-board (Emlid Reach M2), and post-processing was done using a Trimble® R2 antenna (Fig. 3c). The time interval between every shot was 1 s with a frontal and lateral overlap of 85% and 65%, respectively. The GNSS base station is a Trimble® R2 antenna with dual-frequency GNSS tracking, which was set to record data at 1 Hz during the helicopter surveys. The base station and the on-board antenna do not communicate directly, but it is essential that they run simultaneously. The base station was installed close to the center of the region we surveyed, at a maximum distance of 5 km from the region edge. However, the point coordinates were unknown; thus, it was left running between 24 and 36 h to calculate its absolute location for 10 mm-scale accuracy. Using Agisoft Metashape®, we obtained digital elevation models (DEMs) of 8 cm/pixel and orthomosaic images of 4 cm/pixel. We then mapped the surface structures by combining the morphology information from DEMs with optical evidence from orthophotos.

Structural and morphological mapping were carried out manually using QGIS on constructed DEMs and orthomosaic images. Mapped elements were classified into separate vector layers: extensional fractures, faults, vents, buckles, rollovers, and monocline morphologies. The KFS and the central part of the SWRZ are almost free of vegetation, allowing the mapping and straightforward identification of structures, except for the eastern part of the KFS which is densely vegetated. Fracture mapping was undertaken with the intention to mimic the measuring style performed in the field: naturally non-rectilinear fractures were mapped as rectilinear segments that fit the strike of natural features. If a fracture had only minor changes in direction but one main strike, it was mapped as a single straight line with a corresponding strike. If a fracture was composed of two or more segments with different strikes and sizes relevant at the survey scale, each segment was mapped separately when the size was significant compared to the size of the whole fracture. At present, it is not possible to automate this mapping process with satisfactory results.

During our three field missions (2019, 2022, and 2023), we collected structural field measurements along both the KFS and central part of the SWRZ, as well as making ground observations identifying different deformation types across faults (Fig. 2). To better assess the overall fracture kinematics over the entire areas, we systematically analyze the obliquity of open fractures to extract potential kinematic trends. At each station, we measured the strike direction of extensive fractures and opening direction using piercing points and opening lengths (Ruch et al. 2016; Bubeck et al. 2018). The difference between the opening direction and its relative strike direction allows for detecting an oblique-opening component in fracturing and enabled us to estimate right- or left-lateral components during opening (Fig. 2f). Strike measurements were obtained through digital orthophotos and field measurements, while the opening direction and the shear component were estimated only via field measurements. Even though the resolution of orthomosaic images is high enough to identify these features, finding piercing points was usually easier in the field.

We were unable to cover the entire area by foot; thus, high-resolution images were necessary to measure strike variations. It is likely that some non-tectonic or magma propagation features, such as ruptured tumuli, have been included in the imagery mapping, but their influence is minor. To minimize this bias, all the imagery mapping was performed by the same operator.

**Fig. 4:**
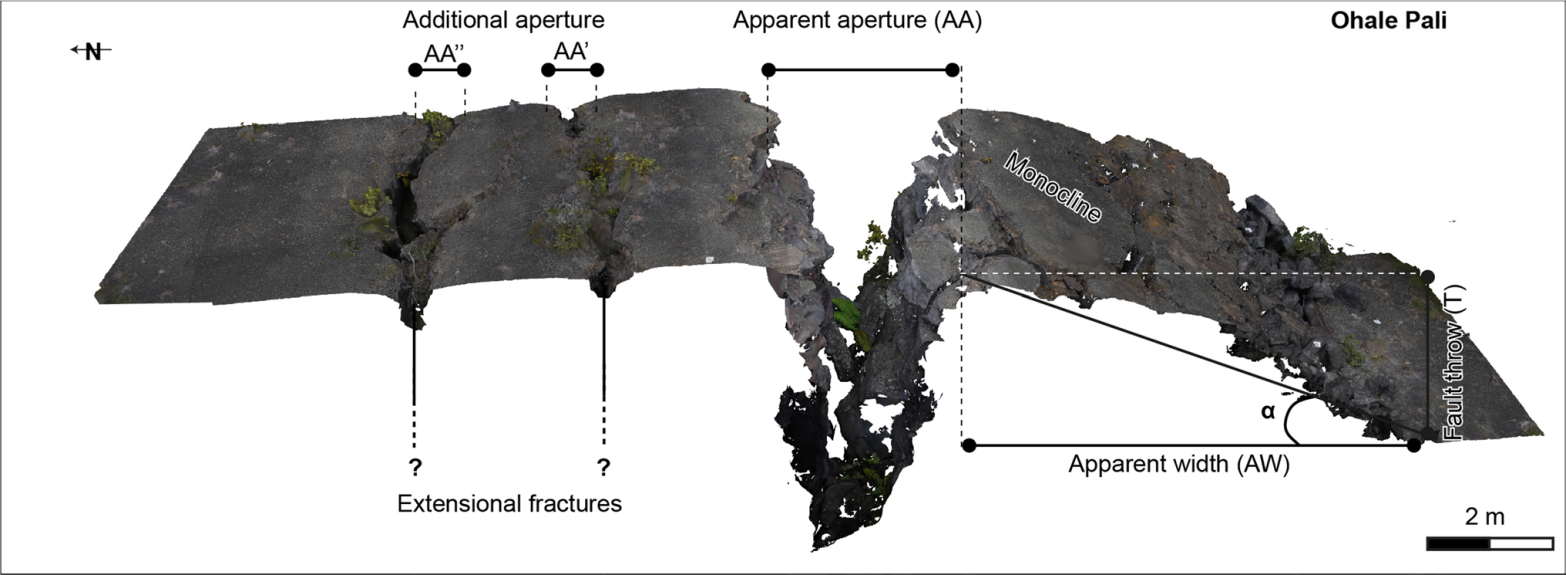
Monocline model characterization from Kettermann et al. (2019) acquired by photogrammetry where AA is the distance between the tilted block and footwall, AA’ and AA’’ are additional aperture of extensional fractures located behind the monocline, AW is the monocline width, AA’ is the extensive fracture, and *α* is the dip of the monocline. See Fig. 9 for location

Morphology identification (e.g., monocline, rollover, extensive fractures) was carried out for some parts of the KFS using field observation, supported by high-resolution DEMs and orthomosaic images. In some cases, we detrend the DEM using a best fit plane to highlight better the morphotectonic structures listed above. These structures were documented for the first time by Duffield (1975) and subsequently by other researchers (Holland et al. 2006; Martel and Langley 2006; Bubeck et al. 2018). However, improvements in image resolution described here have resulted in increased accuracy and identification of surface structures over a broad area of ~ 54 km^2^. We also acquired a few images using a camera fixed atop a 3-m photogrammetry pole to obtain images around specific outcrops for 3D modeling to quantify the measurements of a monocline, rollover, and associated extensive fractures (Fig. 4).

To better assess monocline geometry and estimate vertical and horizontal openings along the main faults, we used a model proposed by Kettermann et al. (2019). Figure 4 shows an example of a typical monocline feature within the KFS. The vertical displacement is given by the difference between the footwall and the hanging wall (*T*). However, the resulting horizontal opening (CA) is estimated using Eq. (1)

1 $${\text{CA}}={\text{AA}}+{\text{AW}}- \frac{{\text{AW}}}{{\text{cos}}\alpha }$$

where AA is the distance between the tilted block and footwall, AW is the monocline width, and *α* is the monocline slope. However, in some cases, we observed extensional fractures located behind the monocline on the footwall (Fig. 4). As such, this opening has been added to our calculation. Results of horizontal opening allow us to estimate the fault dip (*β*) using Eq. (2)

2 $$\beta ={\text{tan}}\frac{T}{{\text{CA}}}$$

## Results

### Morphology observation

**Fig. 5:**
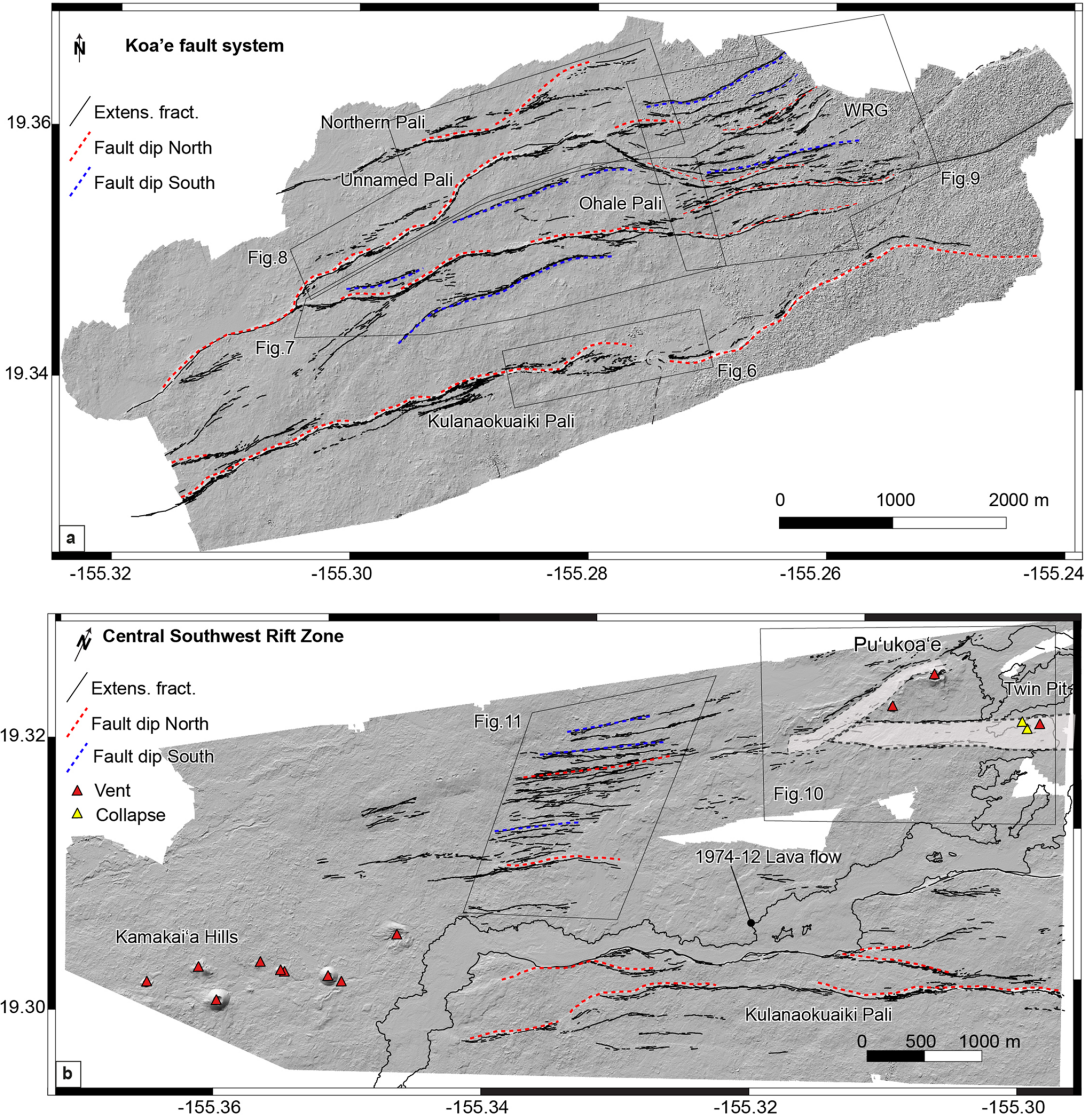
**a** Digital elevation model (8 cm/pixel) over the KFS with extensional fractures (black lines) and faults (dashed red and blue lines). **b** Digital elevation model (8 cm/px) over the central part of the SWRZ, gray polygons represent grabens

The KFS covers a broad area, and we decided to describe a few representative sites that highlight its main structural features. Four main fault segments constitute this zone, but there is no standard classification; thus, we use existing information from the literature and add new terms. From south to north (Fig. 5a), four main faults are observed with the (1) Kulanaokuaiki Pali, (2) Ohale Pali, (3) unnamed pali (Ge et al. 2019), and (4) northern pali (new term). The southern palis are longer than the northern (12 km vs 3 km). In addition to these faults, there is an area referred to as the White Rabbit Graben (WRG in Fig. 5a) characterized by several nested graben and extensional fractures (Swanson et al. 2018).

The second region of interest here is the central part of the SWRZ, where we documented a series of extensional fractures inside a set of grabens (Fig. 5b) and two intersecting grabens south of Pu ‘ukoa ‘e.

### Kulanaokuaiki Pali

**Fig. 6:**
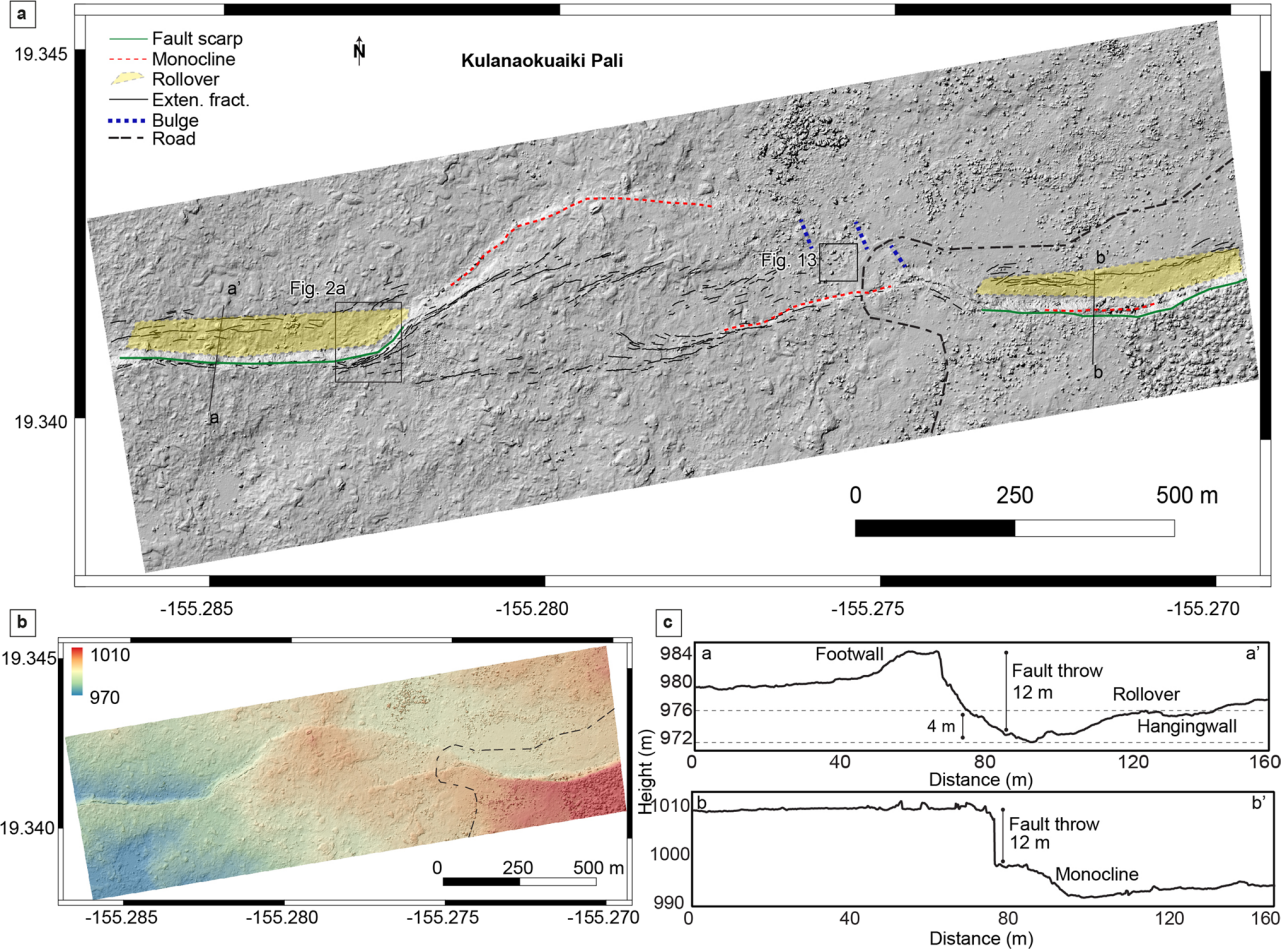
**a** Digital elevation model (10 cm/pixel) over the central part of Kulanaokuaiki Pali at Hilina Pali Road crossing; black lines are extensional fractures, red dashed lines are the monocline fronts, green lines are the fault scarp with sub-vertical offset, blue dashed lines are compressional fold and fault features (see text), and yellow polygons the rollover. **b** Digital elevation model with color gradient showing elevation changes. **c** Topographic profiles across the Kulanaokuaiki Pali

Kulanaokuaiki Pali is the longest fault in the KFS, and it is bordered to the SW by the December 1974 lava flows and by the ERZ south of Maunaulu to the NE. This 12-km-long normal fault represents the southern boundary of the KFS and is crossed by Hilina Pali Road about midway along its trace (Fig. 6). The vicinity of the road crossing provides ready field access to study the pali structure in detail, as highlighted in Fig. 6. East of the crossing, the scarp is as much as 12 m high dipping in a northerly direction. It displaces surficial lava flows of Luamanu that erupted sometime between 1301–1445 CE (Table 1; Fig. S1). The fault strike has a sinusoidal pattern that alternates in the NNE-SSW and E-W directions. Rollover structure is prominent at the base of the scarp, suggesting a shallow listric fault plane geometry. The torn limb of a monocline, representing early development of faulting, lies along the downthrown (northern) side of the main trace and is separated from the main trace by fault rubble.

Farther west (Fig. 6), the main fault trace decreases in throw, and the fault becomes a gentle monocline before disappearing at the surface. The transitional zone is characterized by several ENE segments ordered in a right-stepping sidestep. Kulanaokuaiki Pali reappears as a prominent feature northwest of the Hilina Pali Road crossing, where it offsets surficial lava flows of Āhua that erupted sometime between 1288 and 1388 CE (Table 1; Fig. S1). Continuing to the west, Kulanaokuaiki Pali becomes as prominent as the eastern segment and shows similar structure (14 m offset). In place, the severed monoclinal warp at the base of the scarp is ruptured at the base, where it comes in contact with the hanging wall (downthrown) block. This rupturing corresponds in many places with well-developed rollover structures.

### Ohale Pali

**Fig. 7:**
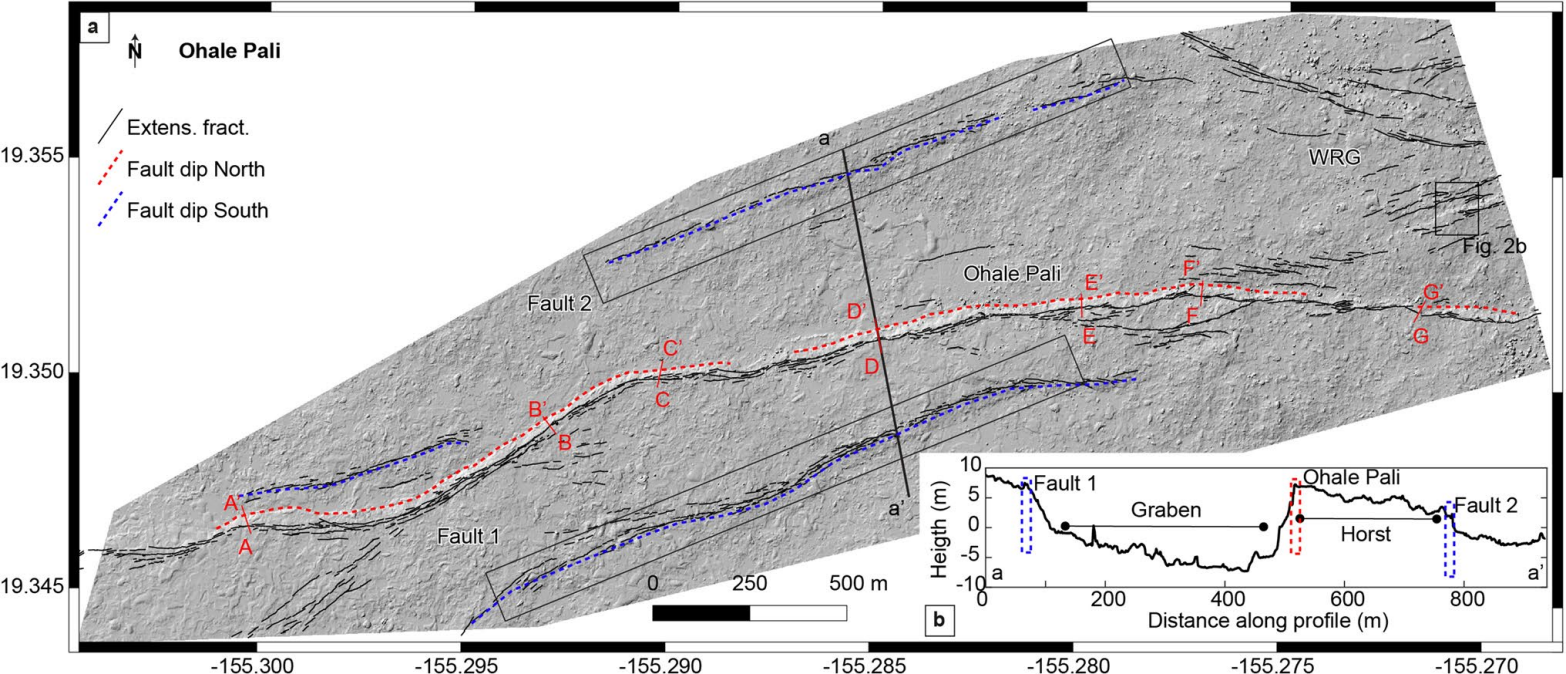
**a** Digital elevation model (10 cm/pixel) over the Ohale Pali (altitude 45° azimuth 315°) where black lines are extensional fractures, red dashed lines are the monoclines dipping north, and blue dashed lines monoclines dipping south. Profiles A-A’, B-B’, C–C’, D-D’, E-E’, F-F’, and G-G’ are located where we applied the monocline model calculation (Fig. 4). **b** Profile across the faults showing monoclines dipping north and souths

Ohale Pali lies a kilometer north of where Hilina Pali Road crosses Kulanaokuaiki Pali (Fig. 5a). It also displaces the same 600–700-year-old lava flow offset by Kulanaokuaiki Pali. It is a ~ 5 km long normal fault oriented N075°, bordered by the December 1974 lava flow to the SW and by the White Rabbit Graben to the NE (WRG in the Fig. 7). Compared to the Kulanaokuaiki Pali, the Ohale Pali has a slightly smaller fault throw (≤ 10 m), dipping steeply to the north. Trending subparallel to Ohale Pali are two parallel fault segments designated Fault 1 and Fault 2, which are characterized by fault throws of a few meters (2–5 m) dipping in the south direction (Fig. 7). These two minor faults are the only southward dipping structures in the central part of the KFS. Together with the two neighboring pali, they form a set of asymmetric horst and graben structures.

The ruptured monocline associated with Ohale Pali has allowed us to measure the apparent opening and the true opening over the fault and estimate fault dip (Fig. 4). Corrected horizontal aperture showed a variation between 1.2 and 3.3 m compared to fault throw that varies between 6 and 8 m. The fault dip estimate varies from 68° to 82° (Table 2).

**Table 2:** Fault parameters estimation for monocline structures at the Ohale Pali. See Fig. 4 and text for parameter calculations (after Kettermann et al. (2019)). See Fig. 7 for profile locations

Profile	Monocline angle (*α*)	Apparent aperture (AA)	Apparent width (AW)	Corrected aperture (CA)	Fault throw (*T*)	Fault dip (*β*)
	[rad]	[m]	[m]	[m]	[m]	[°]
A-A’	0.24	3.1	24.7	2.38	7	71
B-B’	0.30	2.2	16	1.44	6	76
C–C’	0.31	4.4	21.8	3.3	7	64
D-D’	0.40	2.9	19	1.28	11	82
E-E’	0.36	2.2	16	2.61	7	69
F-F’	0.27	3.1	17.8	2.41	6	68
G-G’	0.42	3.6	13.6	2.34	7	71

### Unnamed and northern palis

**Fig. 8:**
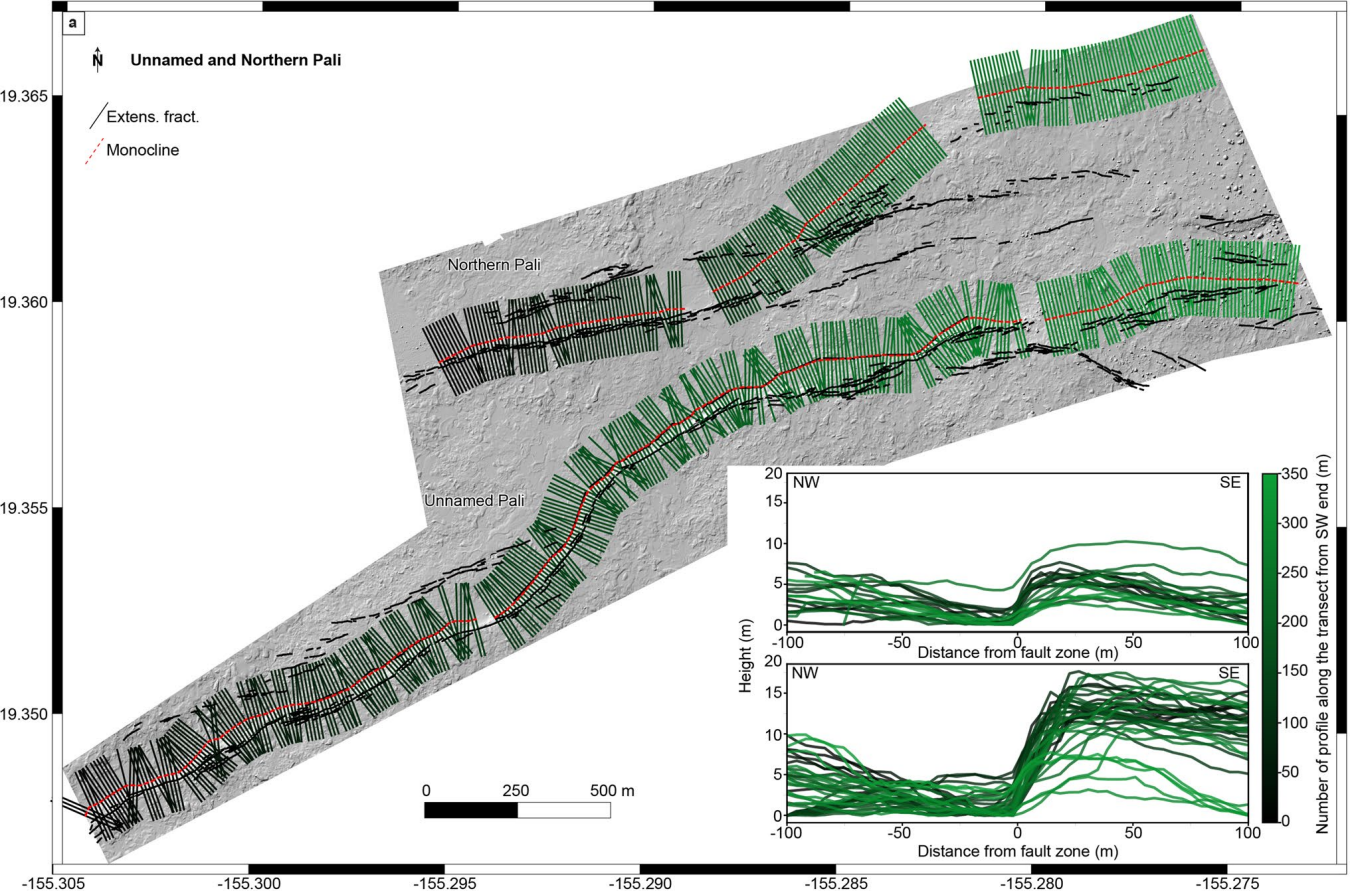
**a** Digital elevation model (10 cm/pixel) over unnamed and northern pali (altitude 45° azimuth 315°), where black lines are extensional fractures and red dashed lines are the monocline fronts. Green scale lines are profiles across the faults (length 100 m) every 10 m. **b** Profiles across the fault showing the lateral variation of vertical offsets

Extensional fractures are numerous close to and subparallel with unnamed pali, like at the Ohale Pali (Fig. 5a). We measured the lateral variations of vertical offset (fault throw) along the unnamed and northern palis by tracing hundreds of transects each with a length of 200 m and spaced from its neighbor by approximately 10 m (Fig. 8). Profiles along unnamed pali show a maximum offset of ~ 17 m with evidence of a rollover. On the other hand, the northern pali shows a maximum offset of ~ 10 m. However, this value may be underestimated because of windblown sand filling rollover depressions (Fig. 2c).

### White Rabbit Graben

**Fig. 9:**
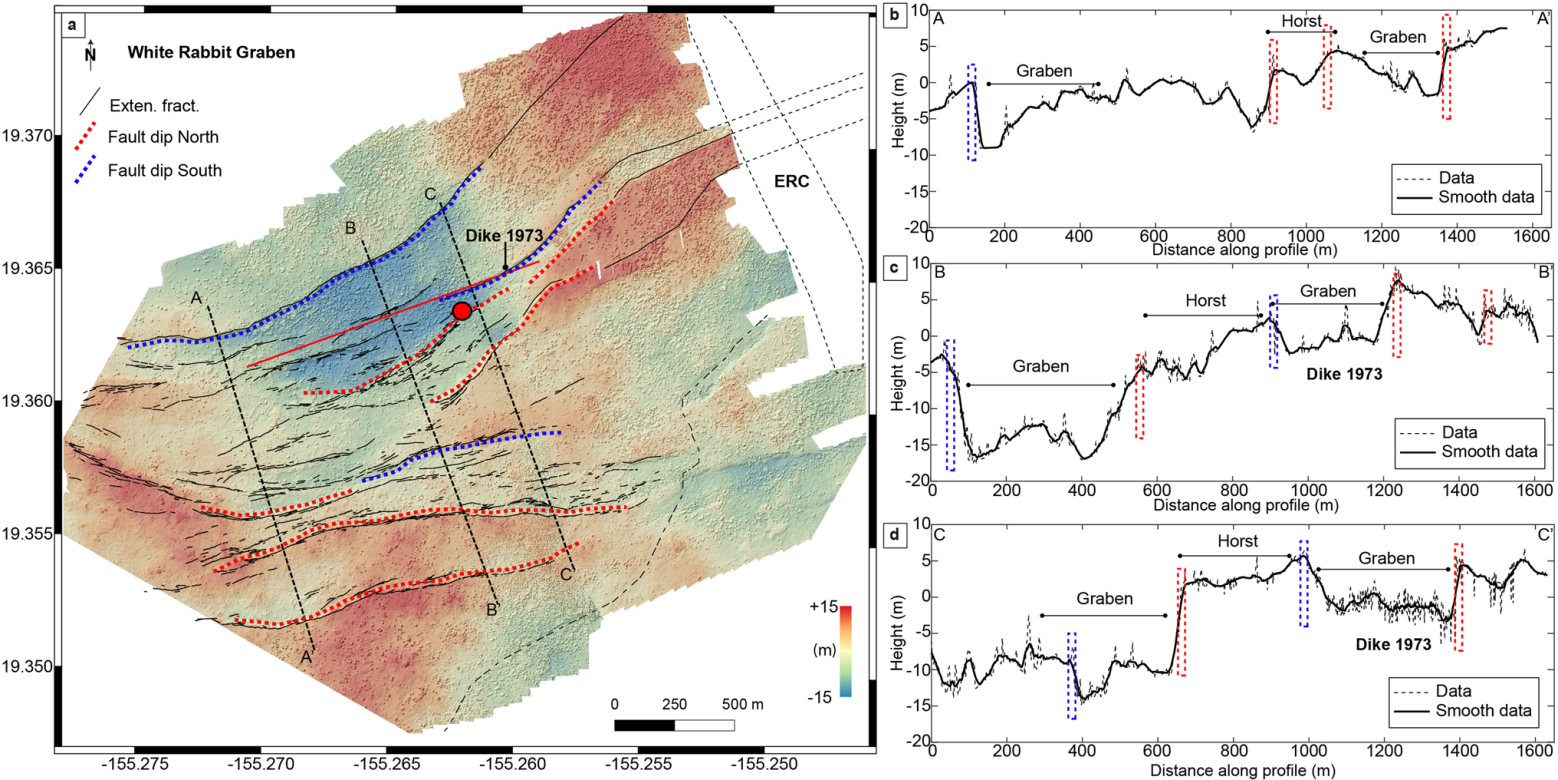
**a** Digital elevation model detrend (10 cm/pixel) over the White Rabbit Graben, where black lines are extensional fractures, and red and blue dashed lines are the faults. The color scale is between − 15 and + 15 to point out the vertical offset. The red line is the dike trace from Swanson et al. (2018). Black dashed lines are profiles across the faults (length 1600 m) that highlight nested grabens. **b**–**d** Profiles across multiple faults that show horst and nested grabens over the region where dashed lines are the data and black lines are the smooth (moving averaged) data. The red circle shows the location of the monocline feature of Fig. 4. See Fig. 5 for location

The eastern part of the KFS is characterized by nested grabens called the White Rabbit Graben (WRG; Podolsky and Roberts (2008). Despite denser vegetation compared to the western sector, high-resolution DEM and LiDAR datasets allow for identifying significant fault traces. These faults reach Chain of Craters Road (the East Rift Connector; ERC, shown in Fig. 9). However, there is evidence of recent lava flows here (Fig. 1) and the trace of the 1973 dike mapped by Swanson et al. (2018). Figure 9 is a DEM (10 cm/pixel) highlighting three main faults dipping north and five dipping south, forming a series of grabens and half-grabens. We also mapped the highest concentration of cracks in our survey over this region (Fig. 9).

### Central part of the Southwest Rift Zone

**Fig. 10:**
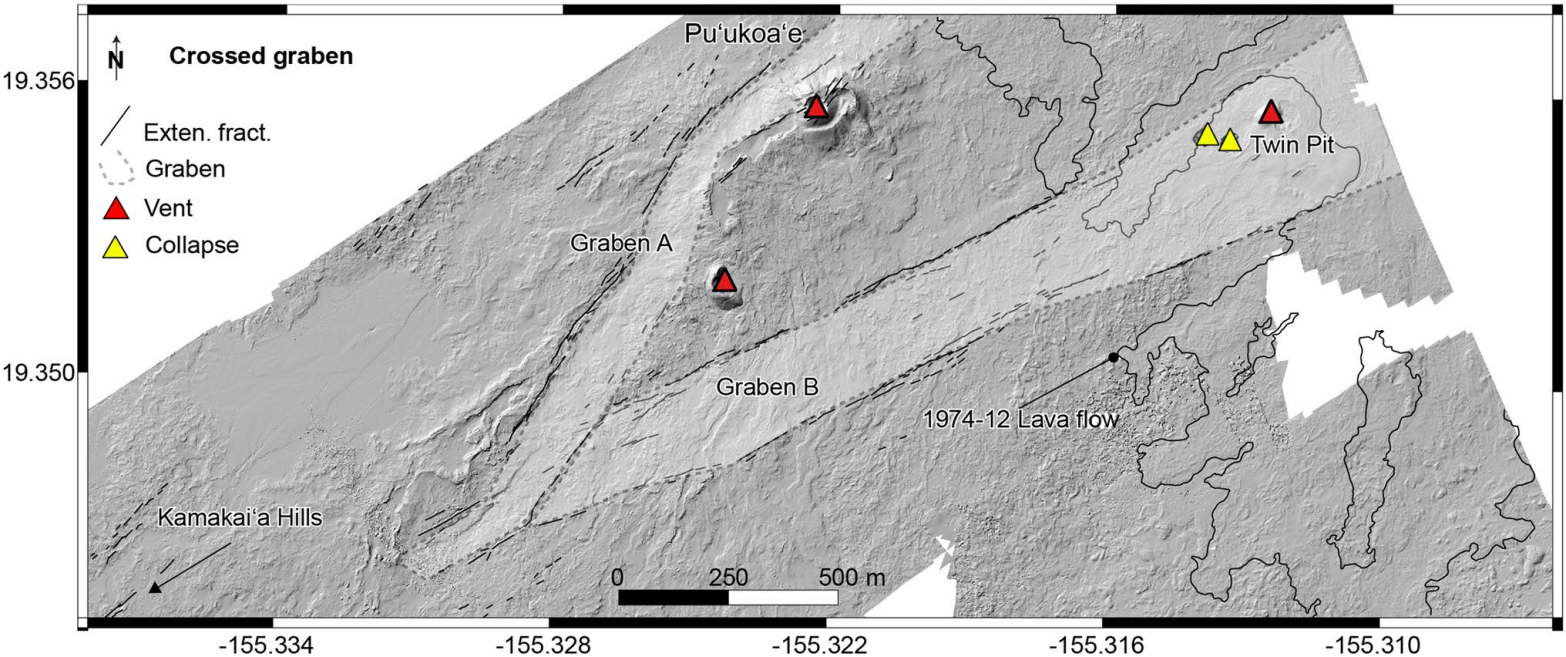
Digital elevation model (10 cm/px) over the central part of the Southwest Rift Zone, where black lines are extensional fractures, the gray polygons are two intersecting grabens, red triangles are volcanic vents, and yellow triangles are collapse features

**Fig. 11:**
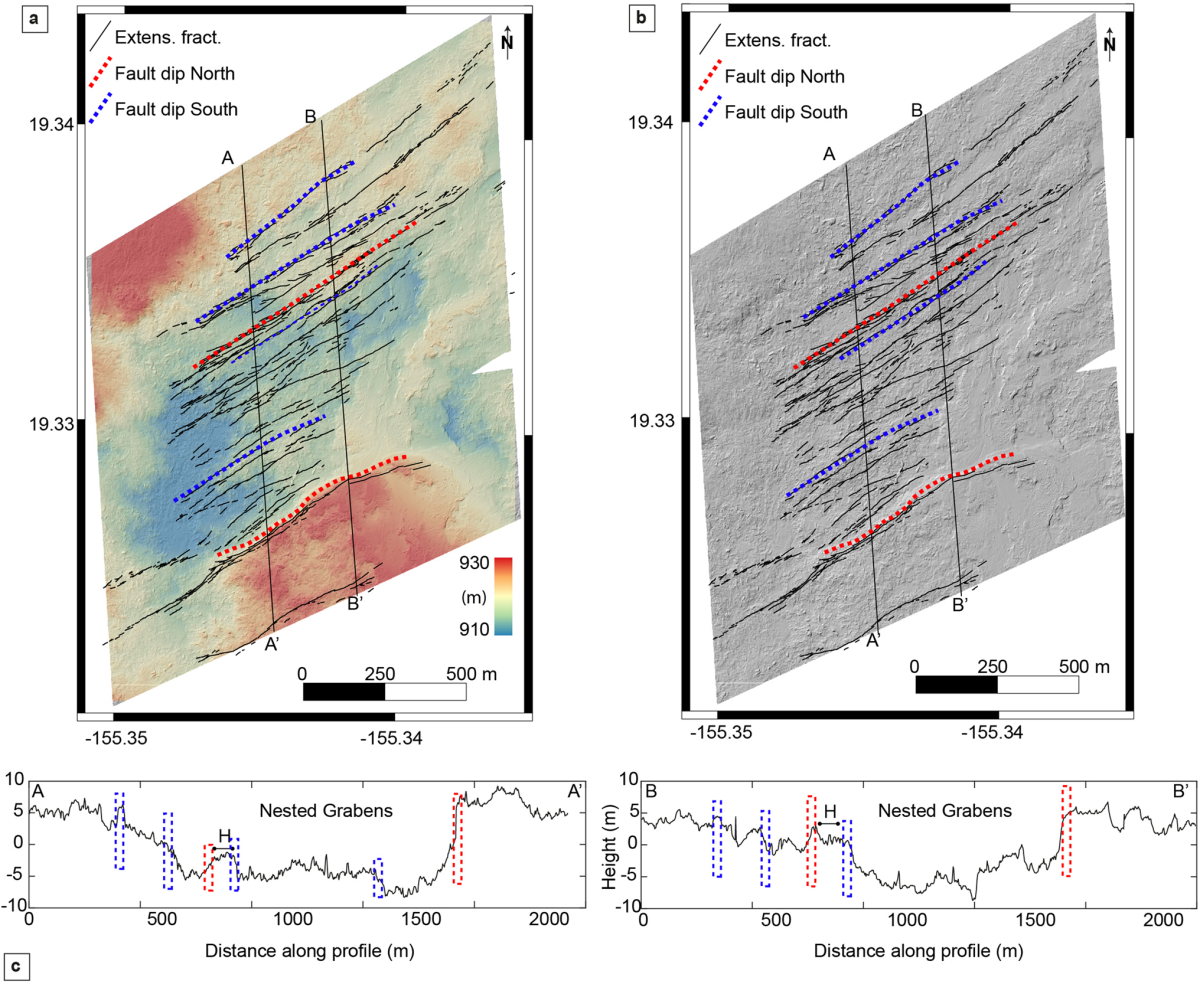
**a** Digital elevation model (10 cm/px) detrended over the central part of the SWRZ, showing multiple nested grabens. **b** Digital elevation model (10 cm/px) over the central part of the SWRZ, showing multiple nested grabens. **c** Profile A-A’ and B-B’ showing nested grabens, horst (H), and faults dipping to the north and south. See Fig. 5 for location

The second area of interest is the central part of the SWRZ (Fig. 5b). This area is smaller than the KFS, but also structurally very complex. This complexity includes two grabens that intersect at an angle south of Pu ‘ukoa ‘e and Twin Pit Craters (Fig. 10). It further continues down-rift and reaches a widespread zone of nested grabens and border faults extending to within about 1 km of the Kamakai ‘a Hills (Fig. 11).

The intersecting grabens have smaller vertical offsets than the systems located within the KFS. Their heights do not exceed 10 m compared to 25 m for the Kulanaokuaiki Pali. The graben southwest of Pu ‘ukoa ‘e (Graben A, Fig. 10) is ~ 120 m wide and 1000 m long, where the north border fault scarp varies between 3 and 5 m high and dips steeply to the SE. The southern border scarp here is less prominent (2–3 m) and dips to the NW. Part of the graben is filled with sand, and a few extensive fractures are visible. Detailed resolution of helicopter survey imagery reveals the other graben, located southeast of Pu ‘ukoa ‘e (Graben B, Fig. 10), which measures ~ 250 m by 2000 m. Graben scarps reach a maximum height of 1.5 m and, in many places, are difficult to trace in the field. This graben is oriented N55E following the orientation of most structures visible over this area. The up-rift ends of both grabens are covered by the December 1974 lava flow from the NE, while to the SW, they are linked with other normal faults and extensional cracks.

The series of nested grabens making up most of the SWRZ structurally is shown by profiles AA’ and BB’ in Fig. 11. Our study area, where this structure is most clearly displayed, measures 1200 by ~ 2000 m. The external fault scarps are the largest ones in this area, with heights as much as ~ 10 m for the southernmost one and 5 m for the northernmost one (Fig. 11). We observed similar nested structures in the WRG, where there is a succession of grabens, horsts, and cm to m-wide extensional fractures.

### Fault mapping and extensional fracture kinematics

**Fig. 12:**
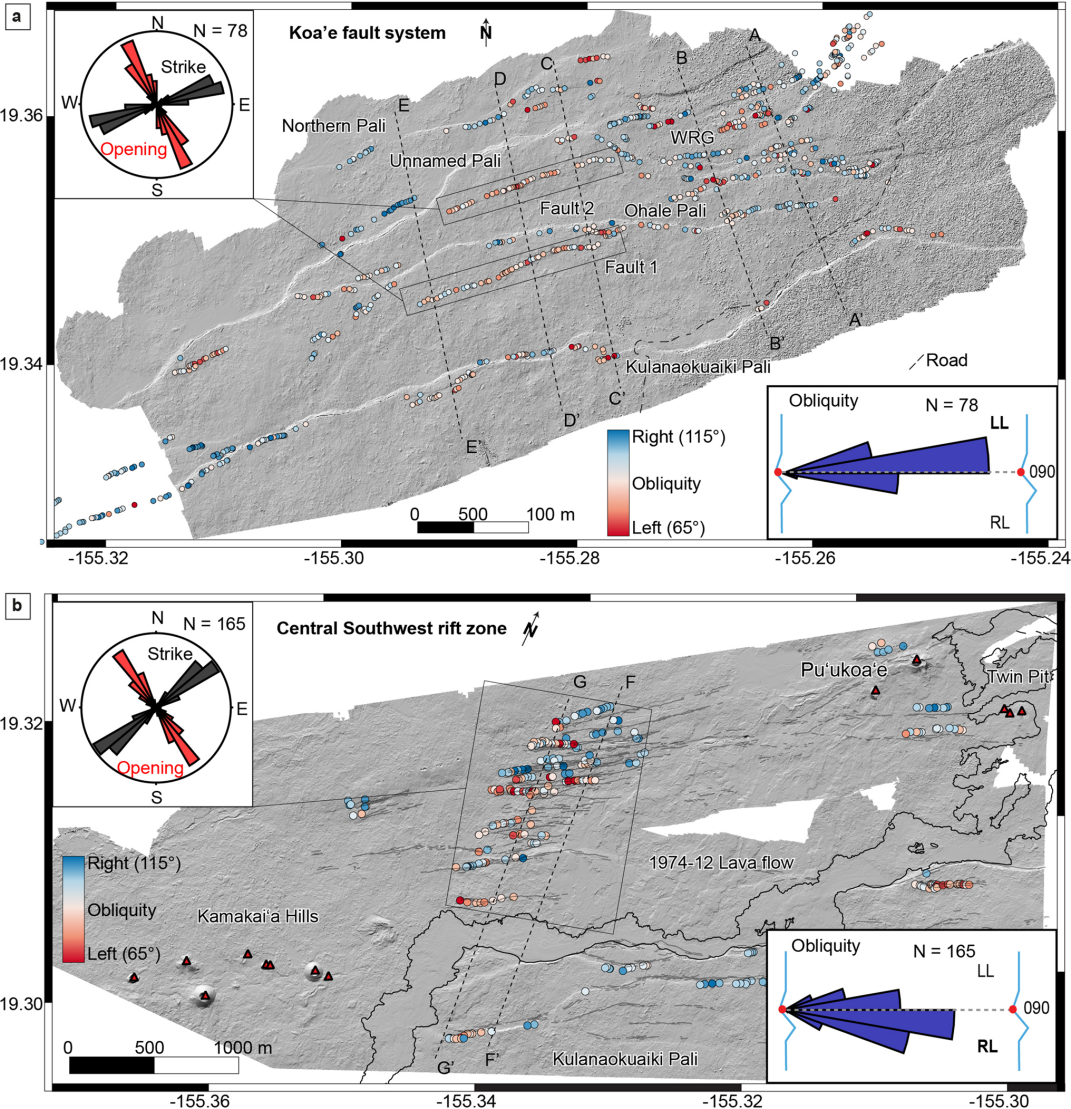
**a** Digital elevation model (8 cm/px) over the KFS with kinematic measurements, where red dots represent left-lateral oblique extensional openings and blue dots right-lateral oblique extensional openings. **b** Digital elevation model (8 m/px) over the central part of the Southwest Rift Zone with kinematic measurements, where red and blue dots represent left-lateral and right-lateral oblique opening, respectively. Rose diagrams showed the strike and opening direction over both areas. Data from the profiles are displayed in Table 3

The KFS and central part of the SWRZ contain thousands of extensional fractures. Here, we look at the overall kinematic trend of extensional fractures to understand the relationship between the central part of the SWRZ, KFS, and ERZ. This serves as a basis for developing a kinematic model of the south flank. Thanks to high-resolution DEMs and orthomosaic images, we measured the orientations of 9436 and 3536 extensional fractures over the KFS and central part of the SWRZ, respectively (Fig. 12). Most of the cracks over the KFS alternate between trends of E-W and NE-SW, with a mean value of N072° ± 0.5. In the case of the central part of the SWRZ, cracks more consistently have a preferential NE-SW (N053° N059°) trend with a mean value of N056° ± 0.5 (Fig. 12).

However, to get more information about oblique-opening associated with ground cracks, we acquired a total of 1127 ground measurements of extensional fractures over both areas. The DEM displayed in Fig. 12 with red and blue dots highlight the oblique-opening of left- or right-lateral motion determined over the KFS and central part of the SWRZ. Values < 90° indicate oblique-opening with a left-lateral component, and values > 90° indicate oblique-opening with a right-lateral component. Measurements of extensional fractures near the Kulanaokuaiki Pali show obliquity in the west, with a maximum value of N115° ± 0.5 and average of N100° ± 0.5, which indicates a slight right-lateral component (Fig. 12a). However, ground measurements acquired over the central and eastern parts of the same pali show a slight left-lateral component, with an average obliquity value of N086° ± 0.5. We also observed two extensional fracture sectors parallel to the Ohale Pali (Fault 1 and Fault 2, above), where we measured 78 cracks showing slight left-lateral component with a mean value of N085° ± 0.5.

**Fig. 13:**
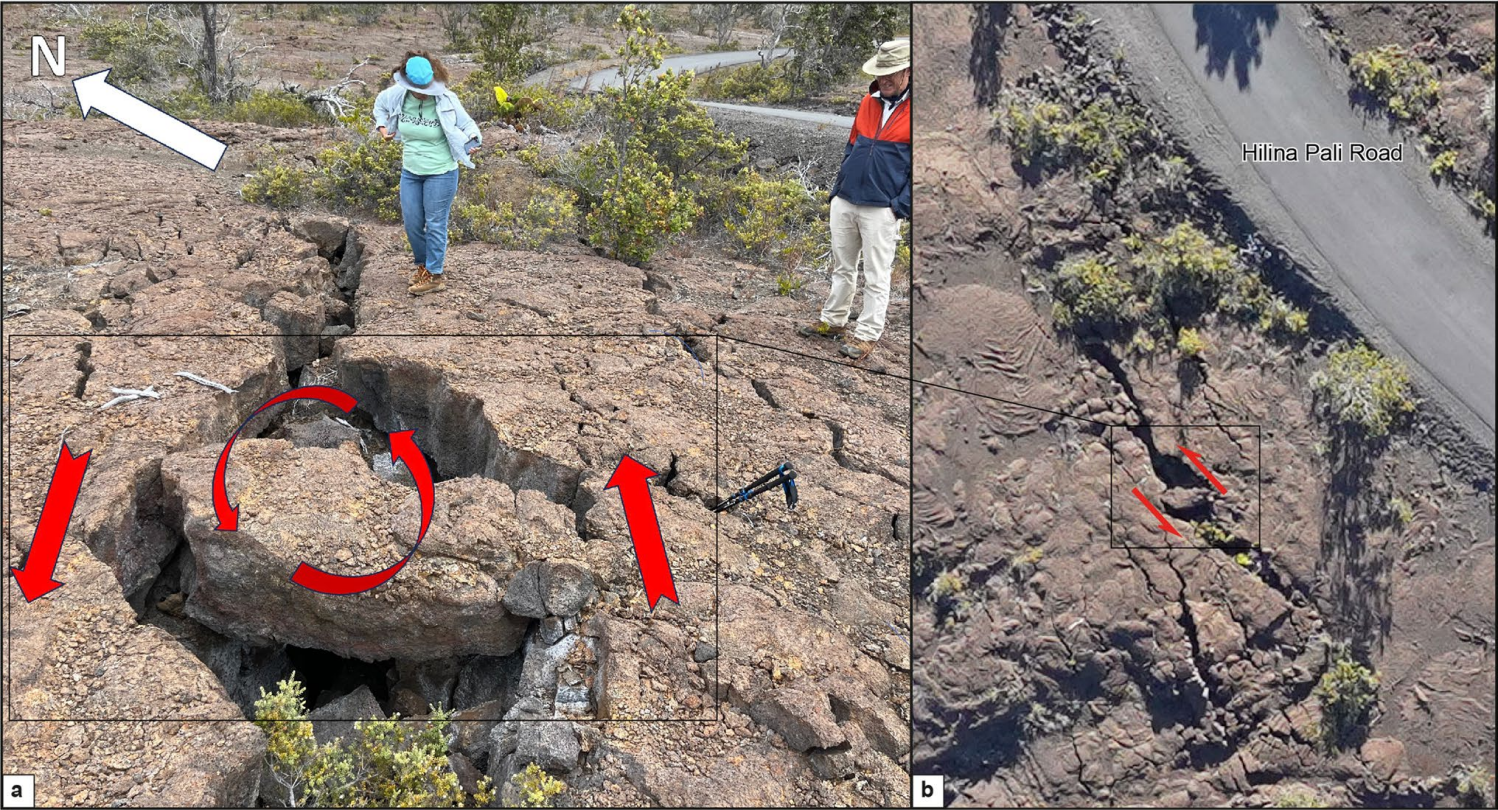
**a** Field picture of a rotated block over the central part of the Kulanaokuaiki Pali. Red lines showing the sense of block rotation evidencing a strong left-lateral component. **b** Optical picture acquired from the helicopter showing the same rotated block close to the Hilina Pali Road. The location is shown in Fig. 6a

The transition zone between the east and west segments of the Kulanaokuaiki Pali fault zone is shown in the center of Fig. 6, in the vicinity of the horseshoe-shaped curve in Hilina Pali Road and the west edge of the area outlined by Fig. 2a. This is best described as a left-lateral transpression zone forming a localized step-over structure. There is strong evidence of left-lateral shear through the center of the zone in the form of an approximately 500-m-long set of obvious right-stepping en-echelon faults (Riedel shears). Each of these faults trends ENE-WSW and they are collectively visible on the DEM of Fig. 6a. Figure 13 shows the counter-clockwise rotation of a block within one of these left-lateral Riedel shears. Opening vectors in the transition zone are dominantly NW. This is consistent with left-lateral motion. At the horse-shoe-shaped curve in Hilina Pali Road, we observed a series of left-stepping en-echelon folds and thrust faults produced by left-lateral transpression. These can be seen on both sides of the road. The east segment of the fault zone crosses the road and forms the southern edge of the transition zone. However, it quickly loses definition due to horsetail splaying within 300 m west of Hilina Pali Road.

The WRG does not show a preferential direction; it shows alternate left- and right-lateral components. Ground measurements acquired over the second study area, the central part of the SWRZ, are less distributed than in the KFS. Most of the cracks measured in the field are in the series of nested grabens (Fig. 12b). Here, we observed a preferential slight right-lateral component with a mean strike value of N094° ± 0.5 except for the easternmost segment of cracks that shows a left-lateral component.

## Discussion

### Cumulative displacement of the KFS and central part of the SWRZ

Our new morpho-tectonic observations over the KFS and central part of the SWRZ highlight the structural diversity of faults, grabens, and extensional fractures in these regions. To understand deformation over the past several centuries in the KFS and central part of the SWRZ, we combine our observations with published ages of lava flows (Table 1) and quantify the long-term displacement over both regions of study. We estimate vertical and horizontal displacements along five profiles in the KFS and two profiles in the central part of the SWRZ (Fig. 12). In the KFS, the five N-S profiles cross four main faults and pervasive areas of extensional cracks. We measured displacement rates using lava flows well-characterized radiocarbon ages.

The profile A-A’ (Fig. 12) crosses lava flows of Āhua that erupted sometime between 1288 and 1388 CE (Table 1) in the WRG. It passes at a near right angle across a series of horsts and grabens and ends south of Kulanaokuaiki Pali. We measured cumulative horizontal displacements of ~ 35 m from eight extensional fractures and faults (Table 3) along this profile line. Overall, we estimate a vertical displacement of ~ 38 m generated by the northernmost fault of the WRG and Kulanaokuaiki Pali. On this basis, we estimate an average displacement rate of 6 and 5 cm/year for vertical and horizontal components in this part of the KFS, respectively (Table 3).

**Table 3:** Displacement rates across the KFS and SWRZ using geochronology and geological data. Cumulative vertical and horizontal displacement rates (cm/year) are measured in both areas. Profile C–C’ is located along the same line measured by Swanson et al. (2018). Profile locations are reported in Fig. 12

Name	# Fault (vertical)	Vertical offset (m)	# Fault + crack (horizontal)	Horizontal offset (m)	Lava flow emplacement ages (calibrated calendar year)	Vertical rate (cm/yr)	Horizontal rate (cm/yr)
A-A’	4	38	8	35	1288–1388 CE	6–5.2	5.6–4.8
B-B’	5	36	11	28	1288–1388 CE	5.7–4.9	4.3–3.7
C–C’	4	32	15	27	1288–1388 CE	5–4.3	4.3–3.7
D-D’	4	37	8	15	1288–1388 CE	5.8–5	2.3–2
E-E’	3	26	13	17	1288–1388 CE	4.1–3.5	2.7–2.3
F-F’	5	23	17	18	1305–1397 CE	3.7–3.2	2.9–2.5
G-G’	5	29	25	16	1305–1397 CE	4.6–4	2.6–2.2

Profile B-B’ (west of profile A-A') is also located in WRG and shows similar displacement rates to profile A-A’ (Table 3). This profile crosses a single lava flow unit, and the displacement rate estimates vary between 5.7–4.9 and 4.3–3.7 cm/year. Moving toward the west, profiles D-D’ and E-E’ are located in the western portion of the KFS, passing through the four palis previously described (northern, unnamed, Ohale, and Kulanaokuaiki). Here, the vertical displacement decreases from ~ 37 m along profile D-D’ to ~ 26 m along profile E-E’. The horizontal displacement is ~ 15 m along profile D-D’ and ~ 17 m along profile E-E’. Both sets of values are lower compared to profiles A-A’ and B-B’ (~ 20 m). Profiles D-D’ and E-E’ also pass through a single lava flow unit (lava flows of Āhua). Here again, the displacement rates vary between 5.8–5 and 4–3.5 cm/year for the vertical and 2.3–2 and 2.7–2.3 cm/year for the horizontal components (Fig. 12a).

In the central part of the SWRZ, profiles F-F’ and G-G’ trend across nested grabens and the December 1974 lava flow (Fig. 10). Here, we measured a vertical displacement of 23–29 m resulting from two boundary faults and a series of nested grabens (Fig. 10). Cumulative horizontal offset is similar to that in the WRG area (16–18 m) with several extensional fractures located close to the December 1974 lava flow (Fig. 12b). The profiles cross the younger lava flows of the Observatory vent from the summit giving a displacement rate of 3.7–3.2 and 4.6–4.2 cm/year for vertical and 2.9–2.5 and 2.6–2.2 cm/year for horizontal components.

Cumulative displacement measured along the profile C–C’ in the central part of the KFS (Fig. 12a) showing 4 cm/year for the horizontal displacement similar with displacement values reported by Swanson et al. (2018) since 1966. Based on extensional fracture measurements, they estimated a horizontal displacement rate of 4.5 cm/year over the KFS between 1966 and 2016 (see Fig. 6 in Swanson et al. (2018) for profile location). We also observed that fault offsets within the KFS decrease from east to west in agreement with their findings. The proximity of the ERZ may play a role in the presence of larger fault offsets over the eastern part of the KFS, as it represents the most active sector of the volcano’s south flank. This is also supported by studies that measured deformation along Kulanaokuaiki Pali associated with historical earthquakes and neighboring ERZ intrusions (e.g., 1965 Christmas activity (Fiske and Koyanagi 1968); 1975 Kalapana earthquake (Lipman et al. 1985); 2007 earthquake (Wauthier et al. 2013); 2012 earthquake (Ge et al. 2019); 2018 eruption (Neal et al. 2019)).

### Magma pathways and kinematic model for the KFS and central part of the SWRZ

**Fig. 14:**
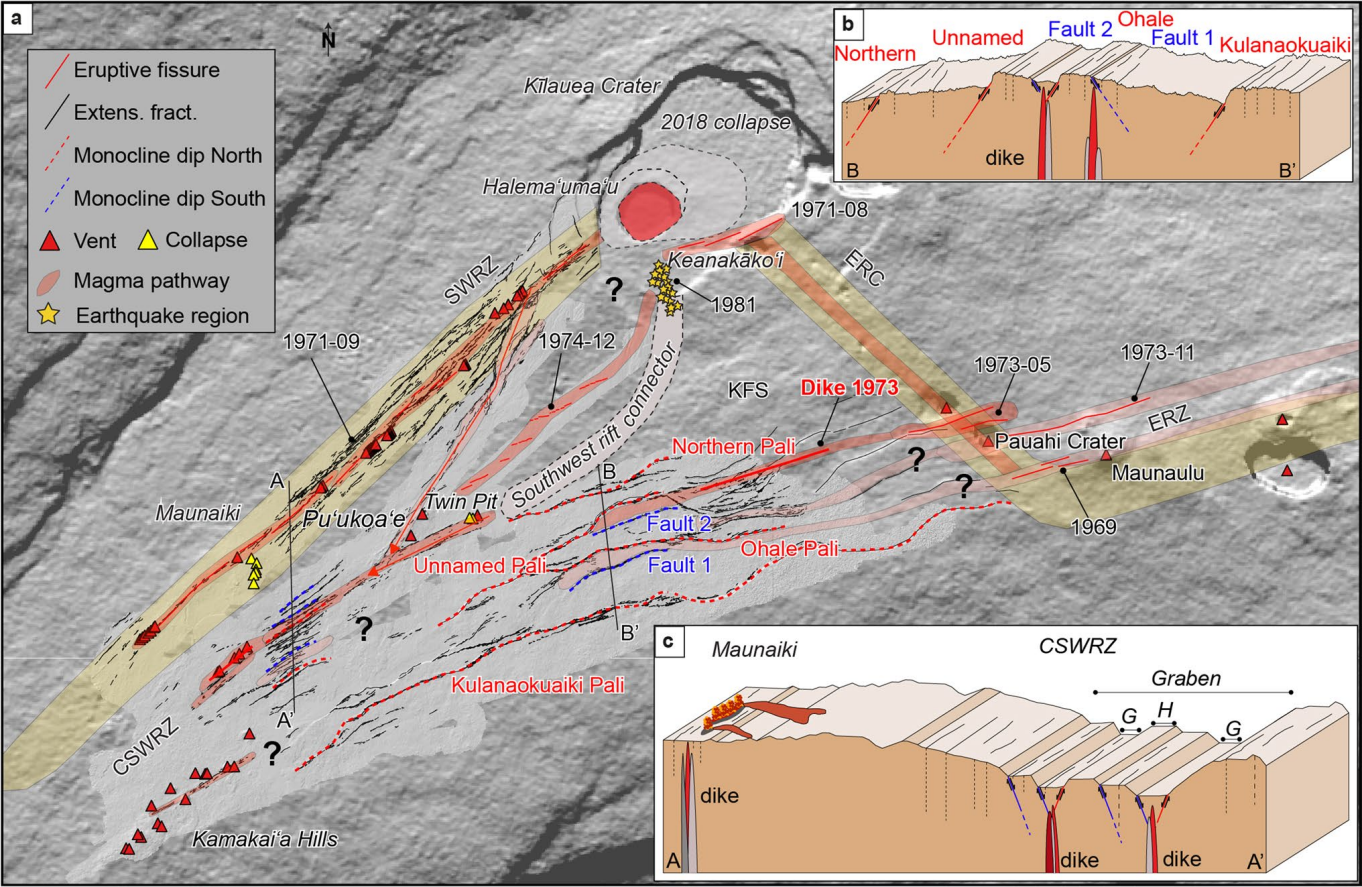
**a** Kīlauea volcano showing the Koa’e fault system and magma pathways. Southwest Rift Zone (SWRZ) and Southwest Rift Connector from Swanson et al. (2018) are a corridor of earthquakes and magmatic intrusions. Orange block represents the evidence of magma pathways and light orange are the potential magma pathways located within the SWRZ and KFS. East Rift Connector (ERC) and East Rift Zone migration have been modified from Swanson et al. (2018). **b** Cartoon of the KFS showing dike intrusions. **c** Cartoon of the central part of SWRZ and Maunaiki showing grabens (G) and horsts (H)

Previous studies (Karpin and Thurber 1987; Delaney et al. 1990; Swanson et al. 2018) discuss magma propagation along both rift zones, yet it is still an open question about the degree to which magma occasionally penetrates pre-existing tectonic faults such as those of the KFS. During an intrusion, magma coming from the southern part of Kīlauea caldera (the “south caldera reservoir”) can follow different pathways (Swanson et al. 2018). A common pathway linking the caldera with the ERZ is through the ERC (Fig. 14). Magma propagates along the ERC and then takes a sharp bend east-northeastward in the area of Pauahi Crater and Maunaulu, following the ERZ where it sometimes can erupt dozens of kilometers from the source summit reservoir, as during the 2018 eruption in the lower ERZ (Neal et al. 2019). Geodetic and seismic evidence indicates that dikes have intruded the eastern portion of the KFS from the ERZ five times in recent decades, primarily from the Pauahi-Maunaulu juncture where the ERC transitions into the ERZ (Swanson et al. 2018). These intrusions have followed the alignment of cracks and faults oriented N072°, subparallel not only to ERZ structures and eruptive fissures east of Maunaulu, but to almost all other eruptive vents and intrusions found at Kīlauea (Fig. 14). Thus, the KFS is intimately connected to the ERZ structural domain, as noted by previous authors (Fiske and Koyanagi 1968; Duffield 1975; Swanson et al. 1976).

We identify a potential magma pathway extending west from Maunaulu along Ohale Pali, and Fault 1 (Fig. 14). A second magma pathway may be related to the westward extension of the November 1973 Pauahi eruptive dike and the dike feeding the nearby November 1979 eruption, which links to the nested graben located in the WRG area (Fig. 14). The location of an inferred dike (Swanson et al. 2018) is reported in Fig. 14, related to a third magma pathway shown by the May 1973 Pauahi-Hiʻiaka eruption extending west to the unnamed pali and Fault 2 (Fig. 14). These observations highlight interpretation of the KFS as a westward extension of the ERZ, forming an integrated structural system. They also explain why overall strain in the KFS is greatest in its eastern part: tectonic structures exist not only as a result of southward flank motion but also the “wedging effect” of frequent intrusions from the adjoining ERZ.

Intrusions within the SWRZ are widely distributed than they are in the eastern KFS and the ERZ where they follow a more localized and mature rift zone (Fig. 14). Evidence of magma migration pathways along the SWRZ is clearer and more widespread than they are in other parts of the study area. As shown in Fig. 14, there are several parallel tracks taken by past magma intrusions, for example, those feeding the September 1971, December 1974, and various prehistoric eruptions (Holcomb 1987; Neal and Lockwood 2003). A narrow zone of seismicity extends southward with the south caldera (Fig. 14). Originally called “the seismic southwest rift zone” by Klein et al. (1987), the zone is more than 2 km southeast of the historically most active alignment of the SWRZ and is could be viewed as having a relationship to the SWRZ much like the ERC related to the ERZ because also likely a response to tectonic extension in the volcano’s southern flank. Swanson et al. (2018) referred to it as the Southwest rift connector (Fig. 14). The only eruption in this area in the last 600 years occurred in December 1974, although earthquake swarms and inflation have continued to occur frequently along this alignment, most recently in January–February 2024 (Poland et al. 2014; Johnson et al. 2015; Nguyen et al. 2022). To the southwest, structures in the central part of the SWRZ are more closely parallel KFS faults, and they might be linked with magma pathways following the westernmost KFS, especially in the area of the Kamakaiʻa Hills (Hazlett et al. 2019). This, however, may suggest a completely through-going intrusive linkage between the ERZ and SWRZ following the KFS (Fig. 14), and there is still no evidence that this exists apart from a minor deposit of prehistoric spatter discovered by Swanson et al. (2018) in the central part of the fault system.

**Fig. 15:**
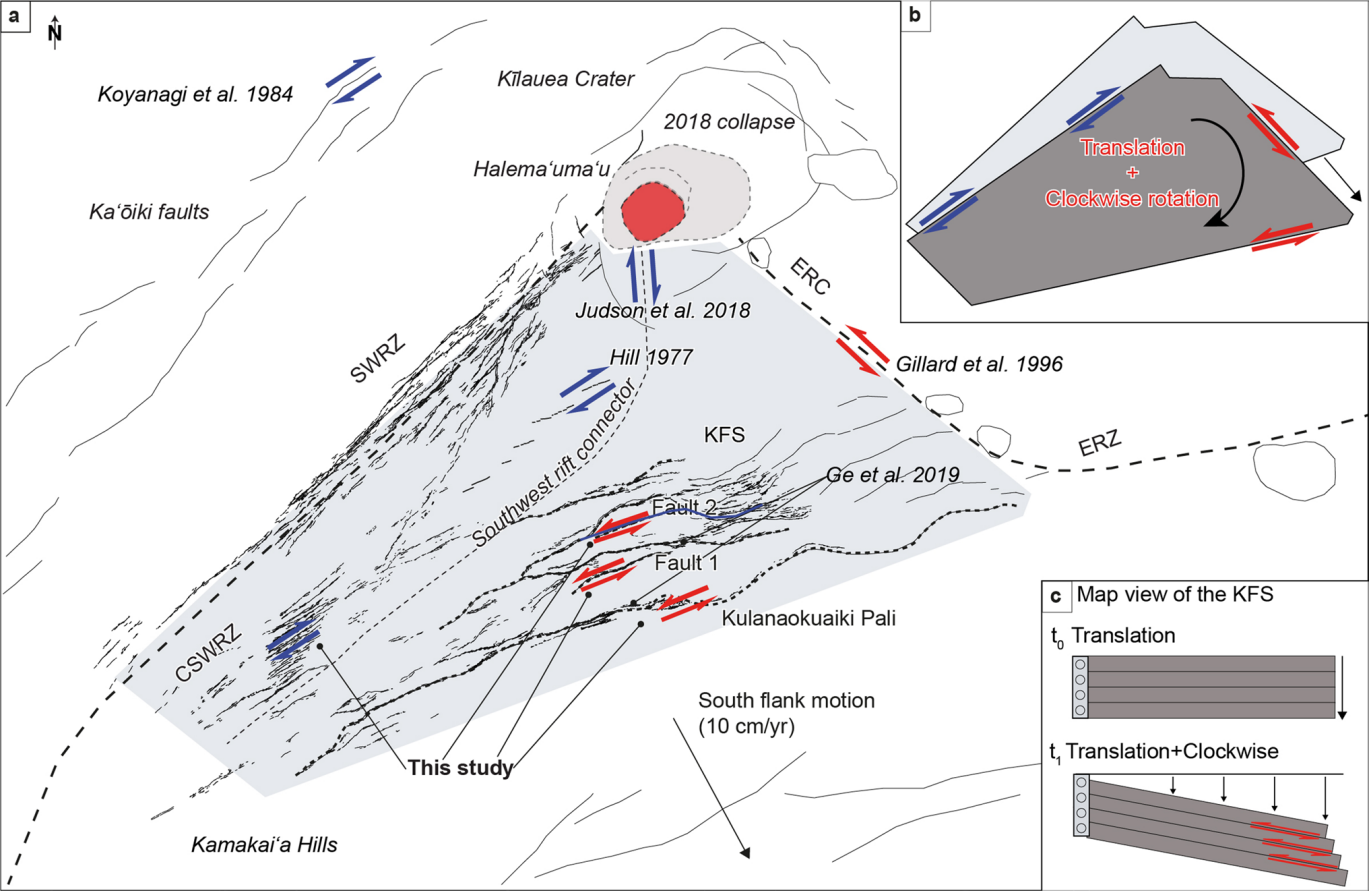
**a** Kinematic model of the triangular wedge of Kīlauea’s south flank. Red and blue arrows indicate respectively the left- and right-lateral component of shear. **b** Cartoon of triangular wedge showing the clockwise rotation and seaward translation. **c** Cartoon showing vector velocity of seaward motion leading to the translation and the clockwise rotation of the KFS

Recent kinematic observations confirm both the seaward movement of Kīlauea’s southern flank and the lateral shear components of movement in the triangular region between the SWRZ, ERC/ERZ, and KFS. Owen et al. (1995) established seaward slip rates up to 10 cm/year for the period 1990–1995 and Miklius et al. (2005) more than 6 cm/year for the period 1997–2004, while Ge et al. (2019) measured a rake of between 074° and 089° during the June 2012 KFS earthquake, indicating normal faulting with a slight left-lateral component (Fault 1 and Fault 2; Fig. 15), consistent with our ground crack measurements and other structures described above (e.g. Figure 13). These new findings are also supported by Gillard et al. (1996) that reported seismic evidence from the adjacent ERC showing left-lateral strike-slip motion (Fig. 15).

Moving to the west into the SWRZ region, seismicity of strike-slip faulting reported by Matoza et al. (2013) during the December 1974 eruption showed a right-lateral sense of shear. This is also indicated by the pattern of en-echelon eruptive fissures (Hill 1977). Similar sense of displacement was reported by Karpin and Thurber (1987) during the 1981 SWRZ intrusion. The northern border of the rift zone is defined by the Kaʻōiki fault system. This structure marks the boundary between Mauna Loa and Kīlauea, two independently deforming volcanic shields. The Mw 6.7 1983 Kaʻōiki earthquake involved a strong right-lateral component (Fig. 15; Koyanagi et al., 1984) which we demonstrate is widely expressed throughout the central SWRZ. Because the magma reservoirs beneath Kīlauea and Mauna Loa deform intensely and frequently, their geographical placement relative to one another helps explain why a pervasive right-lateral shear should exist in the strains observed here. Altogether, these observations support a picture of oblique extensional faulting with a right-lateral sense within the SWRZ that could be explained by regional stress related to the instability of Kīlauea’s south flank (Johnson et al. 2015) plus the orientation of the SWRZ with respect to the major magma reservoirs of Mauna Loa and Kīlauea.

Our work and previous observations also indicate that the entire triangular wedge of crust between Kīlauea’s two rift zones and the KFS is experiencing a slight clockwise rotation over time as slippage pulls it seaward (Fig. 15). Magmatic intrusions that wedge open the crust near the juncture of the KFS with the ERZ contribute significantly to this rotational strain. Individual fault displacements diminish westward along the KFS related to the influence of periodic magmatic deformation events superimposed upon a pattern of persistent tectonic extension.

## Conclusions

Integration of photogrammetry, field observations such as morphological observations, fault mapping, and kinematic measurements are essential to understanding and constraining structural models on volcanoes. These findings, combined with lava flow ages, help to quantify ground displacement over the last seven centuries within the KFS and central part of the SWRZ. Results show a maximum of 6 and 5.5 cm/year of vertical and horizontal displacement, respectively, within the KFS to a minimum of 3 and 2 cm/year within the central part of the SWRZ.

We observe a decrease in fault offsets from east to west in the KFS, in agreement with previous studies (Swanson et al. 2018). This decrease is likely related to the proximity of the ERC and ERZ causing reactivation of faults in the KFS, especially toward the southeast in the Maunaulu area, where magmas intruding from the summit change course toward the east-northeast—the main trend of the ERZ. Magma in some cases intrudes the KFS along multiple pathways occasionally leading to small off-rift eruptions such as that of May 1973.

We also observe that different magmatic pathways are distributed broadly across the SWRZ, in contrast to the ERZ which has very localized dike intrusions. Kinematic observations highlight left-lateral openings over the eastern part of the KFS and slight right-lateral movements for the central part of the SWRZ, accommodating a clockwise rotation of structures that is facilitated by seaward translation in Kīlauea’s south flank (Fig. 15). Likewise, dike intrusion can be stimulated by large south flank tectonic movements, as shown by the May 4, 2018 Mw 6.9 Kīlauea earthquake and ensuing lower ERZ eruptive activity (Neal et al. 2019). Our kinematic results are supported by previous seismic studies (Hill 1977; Gillard et al. 1996; Judson et al. 2018; Ge et al. 2019).

Understanding the relationship between magma pathways and deformation is key to better resolving the links between the caldera, flank motion, and magmatic intrusions. Our fault mapping and kinematic observations provide a high-resolution structural map over the south flank of Kīlauea that is useful for better assessing future dike intrusions and potential eruptions. We now intend to extend the study area further south along the Hilina Pali fault and offshore, integrating bathymetric data, to better understand the entire structure of the Kīlauea’s south flank.

## Postscript

Ongoing dike intrusion in the Southwest Rift Zone, which began on January 31, 2024, has resulted in ground rupture along the grabens described in this work close to the Twin Pit Craters and Pu ‘ukoa ‘e (Fig. 10). At the time of writing (February 2024), we have not been able to evaluate which structures have been reactivated. However, we know that the largest subsidence occurred in this area following the magma pathway described in this publication (Fig. 14).

### Supplementary Information

Below is the link to the electronic supplementary material.

Supplementary file1 (PDF 18322 KB)

## Data Availability

The vector files of the mapped ground cracks, fracture kinematics, Digital Elevation Model, and orthomosaics files are fully available at the following link: https://osf.io/8e5us/. The optical images acquired from USGS server for the 2018 LiDAR mission are available at this link https://hddsexplorer.usgs.gov/.
