# Supplementary figures and images for: Tracking magma pathways and surface faulting in the Southwest Rift Zone and the Koaʻe fault system (Kīlauea volcano, Hawai ‘i) using photogrammetry and structural observations
Source: Bull Volcanol. 2024 Apr 11;86(5):45. doi: 10.1007/s00445-024-01735-7 (PMC11008072; doi:10.1007/s00445-024-01735-7)

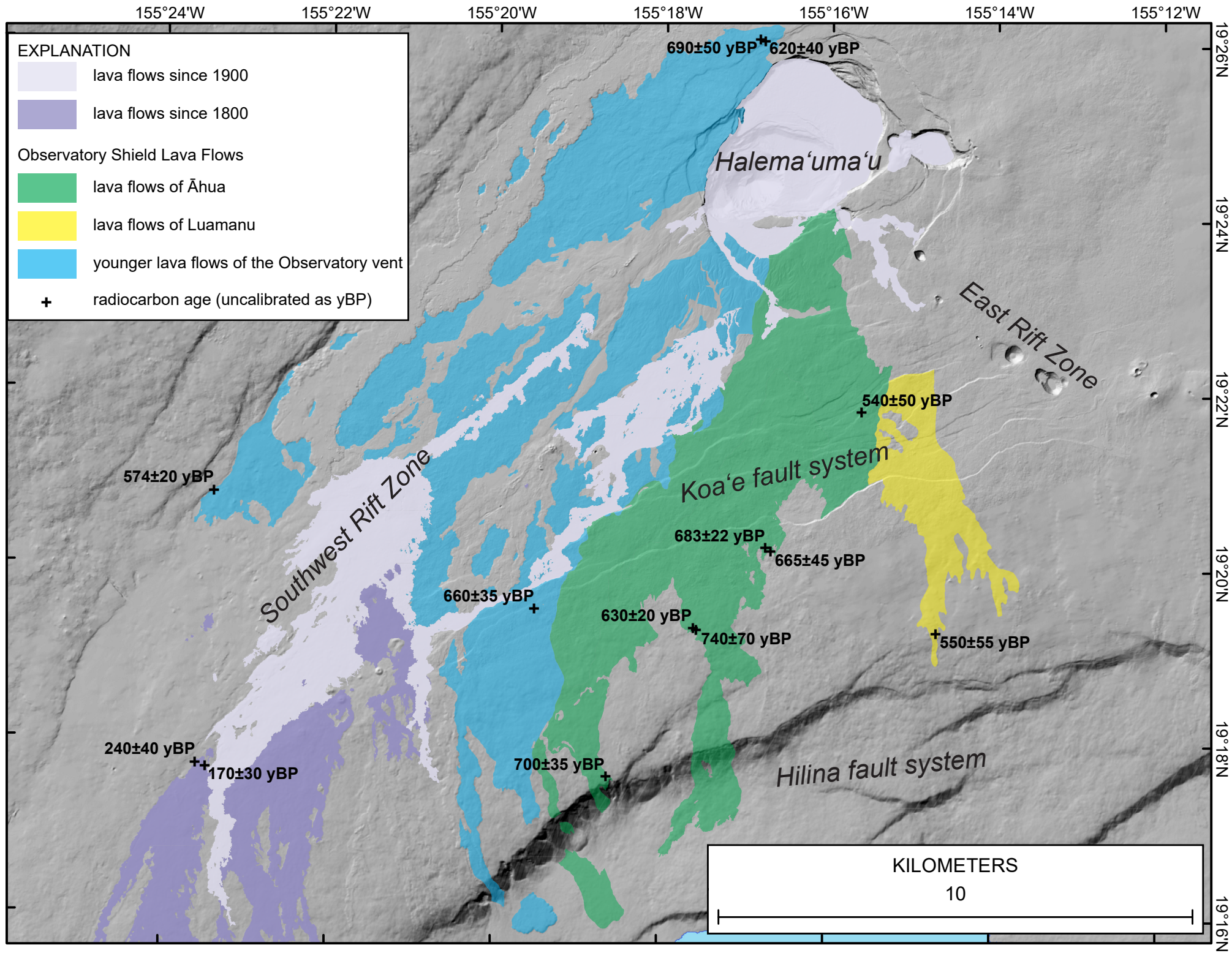

Supplement: Supplementary file 1 — Supplementary file1 (PDF 18322 KB) [file 445_2024_1735_MOESM1_ESM.pdf]
